# SF3B1 mutations in myelodysplastic syndromes: A potential therapeutic target for modulating the entire disease process

**DOI:** 10.3389/fonc.2023.1116438

**Published:** 2023-03-17

**Authors:** Moqin Jiang, Meng Chen, Qian Liu, Zhiling Jin, Xiangdong Yang, Weifeng Zhang

**Affiliations:** ^1^Department of Hematology, First Teaching Hospital of Tianjin University of Traditional Chinese Medicine, Tianjin, China; ^2^National Clinical Research Center for Chinese Medicine Acupuncture and Moxibustion, Tianjin, China; ^3^Graduate School, Tianjin University of Traditional Chinese Medicine, Tianjin, China

**Keywords:** SF3B1 gene mutation, spliceosome, splicing factor mutations, iron metabolism, hyperinflammatory, spliceosome inhibitors, myelodysplastic syndromes (MDS), MDS-RS

## Abstract

Myelodysplastic syndromes (MDS) are clonal hematologic malignancies characterized by ineffective hematopoiesis and dysplasia of the myeloid cell lineage and are characterized by peripheral blood cytopenia and an increased risk of transformation to acute myeloid leukemia (AML). Approximately half of the patients with MDS have somatic mutations in the spliceosome gene. Splicing Factor 3B Subunit 1A (SF3B1), the most frequently occurring splicing factor mutation in MDS is significantly associated with the MDS-RS subtype. SF3B1 mutations are intimately involved in the MDS regulation of various pathophysiological processes, including impaired erythropoiesis, dysregulated iron metabolism homeostasis, hyperinflammatory features, and R-loop accumulation. In the fifth edition of the World Health Organization (WHO) classification criteria for MDS, MDS with SF3B1 mutations has been classified as an independent subtype, which plays a crucial role in identifying the disease phenotype, promoting tumor development, determining clinical features, and influencing tumor prognosis. Given that SF3B1 has demonstrated therapeutic vulnerability both in early MDS drivers and downstream events, therapy based on spliceosome-associated mutations is considered a novel strategy worth exploring in the future.

## 1 Introduction

Myelodysplastic syndrome (MDS) is a clonal hematologic malignancy characterized by ineffective hematopoiesis and morphologic dysplasia, clinically manifested by peripheral blood cytopenia with an associated risk of transformation to acute myelogenous leukemia (AML).MDS is driven by structural chromosomal alterations in neoplastic myeloid cells and somatic mutations caused by tumorigenic and pro-inflammatory bone marrow microenvironment support (1, 2).

In recent years, there have been tremendous advances in the molecular genetic background of MDS. Studies have shown that somatic mutations in the spliceosome gene are present in approximately half of MDS patients and that there is a clear association between specific spliceosome mutations and MDS subtypes (3–5). Among them, Splicing Factor 3B Subunit 1A (SF3B1) mutations are the most common spliceosomal mutations in MDS. The mutational status of SF3B1 is an essential predictor of the presence of ring sideroblast (RS) in the bone marrow (6), occurring in more than 90% of patients with MDS with ring sideroblasts (MDS-RS) ≥5% (7). A large body of supports a high correlation between SF3B1 mutations and disease phenotype (8, 9) and can be used as a disease subtype identifier in MDS (7). In the 5th edition of the world health organization (WHO) classification of haematolymphoid tumours (10), MDS with SF3B1 mutations has been classified as a separate subtype.

Mutations in splicing factors have been shown to occur early in tumor evolution (8, 9, 11), and some investigators believe that it does not have a disease-driving function but rather produces many pro-cancer changes (12). In functional studies of splicing factors, it has been found that these mutations are enriched in dysregulated signaling pathways and cellular processes that induce disease onset and progression and play an essential role in determining the clinical features of the disease (13–15). SF3B1 mutations occurring in MDS result in different splicing alterations that define a disease characterized by RS, ineffective erythropoiesis and low risk of conversion to leukemia, and high overall survival (OS) (5), together suggesting a mechanism of action in MDS.

In this review, we briefly describe the biological functions of SF3B1 splicing factor mutations altered during RNA splicing. We also elucidate the pathophysiological mechanisms of SF3B1 mutations in MDS development and progression, especially regarding disease features of MDS, such as impaired erythropoiesis, dysregulated iron metabolic homeostasis, hyperinflammatory features, and R-loop accumulation. In addition, this review highlights the critical role of SF3B1 as a potential biomarker of MDS in identifying disease phenotypes, promoting tumor progression, determining clinical features, and influencing tumor prognosis, demonstrates the therapeutic potential of targeting SF3B1 splicing factor mutations in MDS and provides new insights into the role of SF3B1 in MDS.

## 2 SF3B1, the core component that drives splicing

RNA splicing is a fundamental process in eukaryotes, which is accomplished by a splicing machinery consisting of five small nuclear ribonucleoproteins (snRNP) and other proteins (16), facilitating efficient mRNA splicing of thousands of genes and playing a crucial role in maintaining cellular homeostasis (17). The spliceosome dynamically assembles on messenger RNA precursors (pre-mRNA) by progressively convening small nuclear RNA (snRNA) and additional protein factors, removing introns (regions that do not encode proteins) from pre-mRNA, leaving exons (the remainder of RNA transcription) attached (18, 19). SF3B1 is a central component of the encoded spliceosome U2 snRNP complex and plays a critical role in recognizing branch point sequences (BPS) and facilitating spliceosome assembly and activation (20).

Early studies performed by second-generation sequencing showed a large number of aberrant selective splicing events in MDS, with somatic mutations in spliceosomal genes present in approximately half of MDS patients (21), with SF3B1 occurring most frequently.SF3B1 splicing factor mutations are thought to contribute to oncogenic transformation, but the underlying mechanisms remain incompletely understood. Mutations in genes involved in the RNA splicing machinery are mutually exclusive. Cells may tolerate only a partial deviation from regular splicing activity, giving a clonal advantage to one type of splicing factor mutant in hematopoietic stem cells (HSCs). This clonal advantage may promote the rate of MDS development and progression (8, 22–25).

Multiple studies have found that the SF3B1 mutation uses an alternative cryptic 3’-splice site (3’Alternative Splice Site, A3SS) during splicing (26, 27), which may be a potential mechanism for its induction of splicing alterations in cancers such as MDS. In addition, events such as retained introns (RI), exon skipping (SE), and mutually exclusive exons (MXE) have also been identified in SF3B1 mutant cells (22, 28). SF3B1 mutation causes MDS to recognize an abnormal 30 splice site (30ss) BPS in the early stages of RNA splicing, causing hundreds of introns to be misplaced and producing abnormal mRNAs with premature translation termination codons (PTCs) (7, 29).

SF3B1 exhibits almost exclusively heterozygous missense mutations in cancer, and its high proportion in hematopoietic malignancies such as MDS occurs within codon 700 (K700E) located in the HEAT domain repeat sequence (22, 30–32). In addition, hotspots such as R625, K666, and H662 also occur in some proportion (9), which are thought to have similar mutational functions due to their spatial proximity to K700E in HEAT repeats (25, 33). SF3B1 mutation-induced degradation of approximately 50% of aberrant mRNAs *via* the nonsense-mediated decay (NMD) pathway results in the downregulation of canonical transcripts and protein expression, and NMD-insensitive aberrant transcripts may also be translated into functionally altered aberrant proteins (28, 34). RNA splicing factors can act as both proto-oncogenic proteins as well as tumor suppressors (35). The occurrence of mutational hotspots and code shift disorders leads to SF3B1 mutations that are considered gain-of-function or tumor morphology-driven mutations rather than loss-of-function mutations. For example, the knockdown of SF3B1 weakens innate immunity, but its induction of abnormal expression of downstream genes contributes to enhanced innate immune signaling (28, 36, 37).

In addition, different functional changes of SF3B1 splicing factor mutations in MDS may produce consistent pathophysiological effects, and the biological consequences show some convergence (28). The current study suggests that the biological consequences of SF3B1 splicing factor mutations may focus on affecting erythropoiesis, iron metabolism homeostasis, high inflammatory environment, and DNA damage to promote the progression of MDS (**Supplementary Figure 1**).

## 3 Pathophysiological mechanisms of MDS with SF3B1 mutation

Many studies have documented that SF3B1 mutations play an essential role in the onset and progression stages of MDS, regulating pathophysiological processes including disorders of erythropoiesis, dysregulation of iron metabolism homeostasis, hyperinflammatory and R-loop accumulation.

### 3.1 Disorders of erythropoiesis

SF3B1 mutant MDS is closely associated with RS morphology and is characterized by ineffective erythropoiesis, often resulting in severe anemia (38). A small sample size study found that an initiating event in the pathogenesis of SF3B1 mutant MDS- RS originates in a rare population of HSCs and propagates the mutation to their myeloid hematopoietic progenitor cells (HPCs) and *de novo* mutations acquired by HPCs at later stages of the disease can confer self-renewal capacity to MDS to drive the transition to leukemia (39).

MAP3K7, a serine/threonine protein kinase with apoptosis-regulating functions in HSCs, was found to be misspliced in SF3B1-mutated MDS patient cells. MAP3K7 is known to be a positive regulator upstream of the p38MAPK signaling pathway. p38MAPK regulates various biological processes, including cell differentiation and apoptosis, and reduced levels of MAP3K7 lead to p38MAPK inactivation. GATA1 is a crucial regulator of erythroid differentiation (40), and disruption of the MAP3K7-p38MAPK pathway directly affects GATA1 function. Meanwhile, the inactivation of MAPKAPK2 and heat shock protein 27(HSP27), potential downstream targets of the MAP3K7-p38MAPK pathway, caused early downregulation of GATA1 and accelerated proliferation, differentiation, and eventual apoptosis of erythroid cells. This study explains the mechanism by which severe anemia occurs in SF3B1 mutant MDS patients and identifies a direct role for MAP3K7 in the proper regulation of erythroid differentiation, leading to further prevention of anemia (41).

Recent findings by Adema et al. show that SF3B1 mutant MDS-RS cells inhibit erythroid differentiation at the terminal stage of erythroid maturation and reduce erythroid release to the PB, leading to the accumulation of terminally differentiated cells in the BM. EIF2AK1 is a metabolic stress-sensitive kinase that binds to its natural ligand heme in the steady state (42). SF3B1 mutations significantly upregulate the EIF2AK1 pathway, resulting in reduced translation of globin mRNA while increasing the expression of the stress response effector ATF4 and its downstream effector DDIT3 to maintain the survival of red lineage cells in heme deficiency.ATF4 and DDIT3 are significant cellular autophagy regulators and control multiple stress stimulus responses, including those affecting iron overload due to abnormal mitochondrial function. Notably, downregulation of EIF2AK1 signaling following the application of hypomethylating agent (HMA) intervention significantly increased the expression of mitochondrial heme biosynthetic enzymes and iron transport proteins and improved terminal differentiation of SF3B1 mutant MDS-RS erythrocytes, suggesting that the development of EIF2AK1 inhibitors could be a transfusion-dependent way to overcome the transfusion dependence of SF3B1 mutant MDS-RS patients as a viable means to overcome the transfusion dependence of SF3B1 mutant MDS-RS patients (43).

Makorin Ring Finger Protein 1 (MKRN1) is a transcriptional co-regulator and an E3 ligase that controls cell cycle arrest and apoptosis by regulating P53 and P21 (44). Huang et al. performed a controlled analysis of purified luciferase knockdown and SF3B1 knockdown red-lineage colony-forming-unit erythroid (CFU-E) cells in human CD34+ cells. Controlled analysis of RNA high-throughput sequencing (RNA-seq) of CFU-E cells revealed elevated protein levels of the P53 gene and its downstream targets P21, BCL2-associated X protein (BAX), and BCL2-binding component 3 (BBC3) and reduced expression of the MKRN1 large isoform. In SF3B1 knockdown red lineage cells, ectopic expression of the MKRN1 large isoform rescued the growth of red lineage cells and restored the protein levels of the P53 gene and its downstream targets P21, BAX, and BBC3, demonstrating that the MKRN1 isoform switch of the P53 pathway may cause the increased apoptosis and cell cycle arrest triggered by the SF3B1 mutation. Their study also showed that SF3B1 knockdown leads to delayed erythroid differentiation and abnormal nucleogenesis in multi-stained and orthostained erythroid cells (32).

In a Gene Ontology (GO) analysis of genes with significant aberrant splicing events in SF mutant MDS, SF3B1 and SRSF2 mutations were found to cause aberrant splicing of mitotic regulators SEPT2 and AKAP8 in CD34+ cells. Moreover, the knockdown of SEPT2 and AKAP8 in human HPCs showed significantly impaired erythroid cell growth and differentiation, indicating the relevance of aberrant splicing of SEPT2 or AKAP8 to erythroid defects in SF3B1 and SRSF2 mutant MDS (23).

Overall, these findings support that SF3B1 mutations in MDS may lead to erythroid defects such as erythrocyte apoptosis, erythrocyte cycle arrest, and delayed erythrocyte differentiation (23, 32, 42, 44), explaining the clinical features of anemia seen more frequently in MDS patients.

### 3.2 Dysregulation of iron metabolism homeostasis

SF3B1 mutations induce aberrant splicing of genes involved in heme synthesis and mitochondrial iron transport, leading to abnormal iron deposition in red lineage cells, resulting in hemoglobin synthesis dysfunction and RS formation. Molecular studies of SF3B1 mutant MDS bone marrow cells revealed that the iron homeostasis regulators ABCB7, TMEM14C, and ERFE were commonly misspliced (23).

Aberrant splicing caused by SF3B1 mutations leading to NMD-induced downregulation of ABCB7 mRNA transcription was demonstrated earlier (45) to underlie the increased mitochondrial iron accumulation found in patients with MDS-RS. Clough et al. constructed an induced pluripotent stem cells (iPSCs) model, which for the first time, effectively differentiated erythroid cells *in vitro* during the formation of RS, recapitulating the pattern of missplicing in MDS-RS patients. Their study confirmed that in SF3B1 mutant MDS, down-regulation of ABCB7 and TMEM14C expression could exert synergistic effects to induce significant up-regulation of ferritin in mitochondria, disrupting the sequence of heme synthesis (46) and reducing the erythrocyte set-up capacity of normal bone marrow cells (47), leading to the formation of RS. In contrast, overexpression of ABCB7 and TMEM14C increased the porphyrin pool required for heme synthesis, repaired the defective erythroid differentiation, and partially rescued the formation of RS. And overexpression of ABCB7 clearly showed a more substantial rescue effect, suggesting that downregulation of ABCB7 expression may be the primary driver of SF3B1 mutant MDS-RS formation (48).

The hepcidin hormone is regulated by the erythroferrone (ERFE) hormone, which regulates iron content, tissue distribution, and iron supply to erythropoiesis in the organism. In SF3B1 mutant MDS patients, a variant protein was found to be generated by induced ERFE transcription in primary SF3B1 mutant bone marrow erythrocytes and, together with canonical transcripts, resulted in ERFE overexpression, maintaining an ability to repress hepcidin transcriptional function. Inhibition of hepcidin by variant ERFE may be responsible for the increased iron load in SF3B1 mutant MDS patients, and the use of hepcidin agonists or targeting ERFE overexpression may provide a potential therapeutic strategy for preventing iron overload and improving erythropoiesis in SF3B1 mutant MDS patients (49).

These studies suggest that SF3B1 mutations alter genes involved in iron metabolism in MDS and are closely associated with heme synthesis and mitochondrial iron transport. Disturbances in iron metabolism make a clinically significant correlation between SF3B1 mutations and MDS-RS and may cause MDS-RS patients to exhibit signs of systemic iron accumulation.

### 3.3 Hyperinflammatory

Increased inflammation is thought to be one of the factors affecting MDS initiation and progression. Inflammatory cytokines are significantly elevated in MDS patients with SF3B1 mutations, impairing the ecotopic function of HSCs and leading to suppression of normal hematopoietic function, which is associated with a poor prognosis (33, 50). Multiple lines of evidence suggest that altered innate immune signaling induced by spliceosomal mutations may alter immune cell function, leading to an increased risk of infection and/or hyperinflammatory features in MDS patients (33, 51, 52). In contrast, suppression of spliceosomal mutations such as SF3B1 may attenuate the risk of inflammation (53). Pollyea et al. found significant changes in pre-mRNA splicing and gene expression in HPCs of MDS patients with SF3B1^K700E^ mutations, upregulating gene expression of several pro-inflammatory signaling pathways, including the pro-inflammatory mediator S100A8 (54).

Lee et al., after shRNA-mediated MAP3K7 downregulation in K562 SF3B1^K700E^ cells and application of the Toll-like receptor (TLR) agonist lipopolysaccharide (LPS) stimulation, found that nuclear factor kappa B (NF-κB) signaling was confirmed to be overactivated at both p-p65 signaling and NF-κB transcriptional activity levels. Furthermore, re-expression of MAP3K7 in K562 SF3B1^K700E^ cells resulted in a significant decrease in p-p65 signaling levels both at rest and after LPS stimulation, indicating that the effect of the SF3B1^K700E^ mutation on NF-κB signaling induction is partially mediated through aberrant splicing of MAP3K7 (53). Pollyea et al. similarly demonstrated that spliceosomal mutations such as SF3B1 enhanced NF-κB activity and LPS-induced production of inflammatory cytokines in macrophages, patient-derived cell lines, and mouse and human bone marrow cells. Interleukin-6 (IL-6) is considered to be an inflammatory cytokine that plays a vital role in inflammation-associated cancers (55). Monocytes from MDS patients with SF3B1 mutations tend to increase the level of IL-6 mRNA, leading to an overproduction of bone marrow cells and abnormal hematological features in MDS patients (33).

Interleukin-1 receptor-associated kinase 4 (IRAK4) is a critical downstream mediator that intimately links the Myddosome complex to inflammatory NF-κB activation. Choudhary et al. found that SF3B1^K700E^ mutation causing exon 6 to be aberrantly retained in MDS samples produced a longer IRAK4-long (IRAK4-L) isoform that maximizes activation of downstream NF- kB signaling. It was confirmed that IRAK4 could serve as a target to alleviate the hyperinflammatory features of MDS (56).

The findings suggest that SF3B1 plays a crucial role in the immune system, leading to an inflammatory microenvironment in MDS through various complex mechanisms that manifest in a hyperinflammatory clinical profile. Most studies have primarily focused on the role of pro-inflammatory cytokines and cellular pathways in MDS with SF3B1 mutations, but further research should also investigate the role of immunosuppressive cells.

### 3.4 R-loop accumulation

DNA replication, recombination, and repair are among the key deregulated pathways in cells expressing mutant SF3B1, and the aberrant formation of R-loops (structures generated by nascent RNA invading DNA) and activation of the DNA damage response lead to increased genomic instability (57).

Aberrant regulation of splicing factors is associated with R-loop formation. On the one hand, alterations in the splicing program may initially impair cellular fitness, causing a compensatory increase in affected cells and contributing to the compensatory accumulation of R-loops in diseased cells (58). On the other hand, aberrant splicing of some genes involved in the repression/regulation of R-loop formation in SF3B1 mutant MDS leads to R-loop accumulation. At the same time, SF3B1 mutations activate the NMD pathway, leading to an increased frequency of mutations in some genes involved in R-loop formation, explaining to some extent the clonal dominance of SF3B1 mutations in MDS (23).

Replication stress and the associated ATR signaling pathway are thought to be critical pathophysiological mechanisms in MDS carrying splicing factor mutations (59). Singh et al. demonstrated that the accumulation of R-loop and associated DNA damage due to SF3B1 mutation activated the ATR pathway involved in DNA repair in MDS cells. They found that inhibition of the R-loop rescues cellular defects, including DNA damage and ATR-Chk1 activation, and that this mechanism is preferentially sensitive to the Chk1 inhibitor UCN-01. UCN-01 promotes apoptosis and kills tumor cells, suggesting that Chk1 inhibition alone or in combination with splicing factor modulators may be a novel therapeutic strategy for targeting splicing factor mutated cells. Preclinical evidence is provided for the possibility that MDS patients with SF3B1 mutations may benefit from Chk1 inhibition, thereby exploiting the R-loop-related vulnerability induced by these mutations (37).

SMG1 is the core kinase that activates the NMD machinery in animals (60). Cheruiyot et al. showed that in cells with mutations in the spliceosome gene SF3B1, SMG1 activity is suppressed, and the formation of toxic protein products may lead to the accumulation of R-loops, further leading to impaired DNA replication, DNA damage, chromosomal instability, and cell death. In contrast, R-loop accumulation in the genome can be rescued by overexpression of the endogenous nuclease RNAse H1, which promotes RNA breakage in RNA-DNA heteroduplexes, suggesting that R-loop is a powerful potential mechanism for synthetic lethality between SF3B1 mutation and NMD disruption. Thus, inhibition of NMD, which more clearly can be considered as inhibition of SMG1, may be an attractive target for the treatment of SF3B1 mutant MDS (30).

SF3B1 mutations trigger partial A3SS events resulting in code-shifting mutations, leading to reduced generation of stop codons and downregulation of the NMD pathway. In contrast, RI events can activate NMD or increase mRNA stability. Pellagatti et al. analyzed events with aberrant splicing in MDS with SF3B1 mutations, and more than half of the A3SS events also exhibited reduced RI. They found that loss of XPB activity, an essential component of the encoded transcription factor IIH (TFIIH) (61) with eukaryotic transcriptional and DNA repair roles, promotes R-loop-mediated DNA damage. The use of upstream A3SS in the upregulated intron would insert six amino acids between the decapping enzyme and ResIII structural domains, potentially altering the expression of disease isoforms and affecting protein levels and function. It is suggested that reduced RI events may be associated with the occurrence of upstream A3SS in SF3B1 mutants (23).

In conclusion, SF3B1 mutations critically impact the onset and advancement of MDS by causing genomic abnormalities through the production of aberrant R-loops and/or compensatory R-loop accumulation. These discoveries on R-loop accumulation and DNA damage offer fresh perspectives on the underlying mechanisms of MDS and may pave the way for groundbreaking approaches to managing the disease.

### 3.5 Other mRNA defects

Missplicing leads to defective mRNA production and disruption of cellular pathways, with significant dysregulation of pathways critical for translational regulation in SF3B1 mutant MDS (23), which may affect the levels of mRNA species in the cytoplasm and, consequently, cytoplasmic homeostasis (62).

The SUGP1 spliceosomal protein plays a vital role in recognition of spliceosomal branching sites, and its defective interaction with the mutant SF3B1 leads to the use of cryptic A3SS. Zhang et al. conducted a series of studies on the interrelationship between SUGP1 and SF3B1. They found that the level of SUGP1 was significantly reduced in the SF3B1 mutant complex, while the level of SUGP1 mRNA was substantially increased in SF3B1. It indicates that the defective interaction between SUGP1 and SF3B1 mutations during mutant spliceosome assembly may alter the normal splicing function and induce oncogenic changes. Mutant cells compensate for defective SUGP1 by an autoregulatory mechanism that increases SUGP1 levels, and overexpression of SUGP1 drives the protein into the mutant spliceosome, partially rescuing disease-driven splicing changes and suggesting the possibility of therapeutic intervention (24, 63).

Liberate et al. established an isogenic cell model of SF3B1^K700E^ using CRISPR/cas9 genome editing technology. They found that mRNAs encode transport RNA (tRNA) synthase and ribosomal components were defective in SF3B1^K700E^ mutant cells, resulting in deficient tRNA synthesis and increased spliceosomal components. Meanwhile, steady-state protein synthesis was not significantly affected, possibly through upregulation to maintain the clonal advantage of SF3B1 mutant cells in splicing-deficient tumors. The nucleoside analog 8-azaguanine is known to bind to both ribosomal RNA (rRNA) and tRNA, targeting both RNA metabolism and splicing. SF3B1 mutations resulting in simultaneous defects in splicing and tRNA/ribosome stability may expose a unique vulnerability to dual targeting using 8-azaguanine, providing a therapeutic opportunity for MDS with SF3B1 mutations (63).

In conclusion, SF3B1 mutations severely impact mRNA levels in MDS. They may even disrupt the translational pathway, leading to a dominance of MDS in the clonal nature of the tumor. However, the specific role of many defective mRNAs in MDS still needs to be fully understood. Conducting a more in-depth study in the future will provide a more comprehensive understanding of the role of SF3B1 mutations in MDS.

## 4 Therapeutic strategies for MDS targeting SF3B1 mutations

### 4.1 Treatment overview

The NCCN guidelines for the management strategy of MDS are based on risk stratification by the International Prognostic Scoring System (IPSS and the revised IPSS-R) and the WHO Prognostic Scoring System (WPSS), which classify patients with MDS into low-risk and high-risk categories (64). Given the high correlation with the MDS-RS entity, MDS with SF3B1 mutation is mainly seen in the low-risk category (65, 66). The main feature of low-risk MDS is the hematocrit, and management strategies are focused on improving anemia or thrombocytopenia, reducing transfusion requirements, improving quality of life, attempting to prolong overall survival, and reducing the risk of AML transformation (67). The management strategy for MDS with SF3B1 mutations is similar to that of low-risk MDS in patients who are extensively treated with erythropoietin (EPO) for chronic anemia, receive regular red blood cell transfusions, and are relapsed or refractory after erythropoiesis-stimulating agent (ESA) therapy. Lenalidomide, hypomethylating drugs, and immunosuppressive therapy are the only remaining treatment options for most patients, but durable responses are limited, and these drugs may carry significant adverse effects (68, 69).

The advent of drugs such as Luspatercept has addressed some of the unmet needs of these patients. Luspatercept, a novel fusion protein, binds to erythropoietic transforming growth factor beta (TGF-β) and promotes *in vivo* and *in vitro* red lineage cell differentiation by reducing intracellular levels of reactive oxygen species without any significant effect on iron homeostasis and mutant allele load (70). Luspatercept is well tolerated and effective in the treatment of anemia in low-risk MDS and is approved for the treatment of transfusion-dependent low-risk MDS patients with RS and/or SF3B1 mutations (7, 71–74). In addition, recent studies have found that Luspatercept has the ability to stimulate osteoblast maturation and may improve both MDS with anemia and bone loss (75).

Data suggest that patients with MDS with SF3B1 mutations may respond better to erythropoiesis-stimulating agents (ESAs) than those without SF3B1 mutations (76). In addition, MDS patients with SF3B1 mutations may also respond better to treatment with lenalidomide (LEN) compared to other high-risk mutations (e.g., ASXL1, U2AF1, TP53, etc.) (77). It has been found that MDS patients with both SF3B1 mutations and del(5q) are more similar to isolated del(5q) MDS in terms of immunophenotypic characteristics in the red and ancestral lineages and may benefit more from lenalidomide than Luspatercept in first-line therapy (78).

Disease-related complications (i.e., hemocytopenia) are the leading cause of death in most low-risk MDS. While Luspatercept effectively improves anemia in low-risk MDS, managing thrombocytopenia and neutropenia is challenging. The low-risk disease can be inert, and despite aggressive treatment, mild thrombocytopenia disrupts the quality of life and exacerbates potential comorbidities in patients (79), perpetuating the high mortality rate in MDS. Therefore, effective therapeutic targets and new treatment strategies are urgently needed.

Increasing evidence that knockdown or exogenous overexpression of SF3B1 mutations regulates tumorigenesis and progression of MDS suggests that SF3B1 represents a potential therapeutic target, and there is an urgent need for appropriate therapeutic strategies. Several therapeutic strategies targeting SF3B1 have been reported to treat MDS in recent years, broadening the scope of SF3B1 as a “drug” target. Strategies targeting SF3B1 have shown higher specificity and efficiency than therapeutic strategies for low-risk MDS. Although preclinical studies have shown a high degree of feasibility, there are still many barriers to achieving these approaches, such as limited efficacy, poor safety, or studies not designed to select sensitive patients. Therefore, before these treatment strategies can be genuinely applied in clinical practice, there is still a long way to go.

### 4.2 Treatment of targeted spliceosomes

Compared to some hematopoietic malignancies without spliceosomal mutations, those with spliceosomal mutations are more susceptible to additional splicing perturbations *in vivo* (80). Given the preferential dependence on splicing function in MDS-associated cells with RNA splicing factor mutations and the progressively more prominent pathogenic role of SF3B1 spliceosomal mutations in MDS and cancer, interest in the pharmacology of targeted splicing has been stimulated.

Small molecule spliceosome inhibitors have great potential as a new therapeutic approach in cancer treatment. Several novel compounds (**Supplementary Table 1**) (81–85) targeting the RNA splicing machinery have been identified, such as pladienolides (E7017), derivatives of FR901464 (spliceostatin A, meayamycin), Herboxidiene (GEX1A) (86), Jerantinine A, OTS964, and H3B-8800, which interfere with the spliceosome complex to target specific steps in spliceosome assembly and/or catalysis. However, these drugs have not yet entered clinical use. Ensuring overall safety in patients is more complex, and some compounds have even proven challenging to synthesize (24).

E7107, a derivative of pladienolide B, disrupts the assembly of U2 snRNP at the 3’ splice site by impairing the ability of U2 snRNP to bind pre-mRNA (87). Unfortunately, optic nerve damage was noted in the Phase I clinical trial, so further studies of this compound were put on hold (88). Spliceostatin A (SSA), a methylated derivative of FR901464, has a similar mechanism of action to that of E7107. It inhibits *in vitro* splicing and promotes pre-mRNA accumulation by binding to the SF3b protein complex (89). Jerantinine A is a novel indole alkaloid with potent anti-tumor cell proliferative activity by inhibiting microtubulin polymerization, upregulating SF3B1 and SF3B3, and inducing G2/M cell cycle arrest and tumor-specific cell death (90).

Several of the aforementioned small molecule spliceosome inhibitors are still a long way from clinical application due to unacceptable adverse effects or difficulties in synthesis (91). H3B-8800, as a very promising oral SF3b complex modulator, can preferentially kill blood and epithelial tumor cells with spliceosome mutations (76), showing satisfactory results in both safety and clinical efficacy in phase I clinical trials in patients with advanced myeloid malignancies. They also found that H3B-8800 may be able to inhibit abnormal splicing of TMEM14C in MDS patients (92), which may have better efficacy in patients with MDS-RS. Besides, OTS964, a highly selective CDK11 inhibitor, was recently suggested to block the critical step of SF3B1 spliceosome activation and lead to extensive RI events and accumulation of non-functional spliceosomes on pre-mRNA and chromatin (87).

### 4.3 Treatment of downstream events of targeted splicing mutations

A high percentage of splicing events occur in SF3B1, allowing the gain or loss of coding RNA sequences or the introduction of frameshift changes to generate new protein linkages, thus generating predictable new coding sequences. Therapeutic approaches targeting predictable downstream events are being explored (29). To date, a large number of studies have found that SF3B1 mutations exhibit therapeutic vulnerability in terms of functional defects (24, 32, 37, 42, 49, 57, 63). We previously mentioned that SF3B1 mutant MDS often undergoes aberrant activation of the DNA damage response and that this genotoxic damage can be repaired using DNA damaging agents (e.g., etoposide) or synthetic lethal small molecule inhibitors (e.g., PARP inhibitors) by selective targeting of tumor cells carrying SF3B1K700E mutation is accomplished by selective targeting of tumor cells carrying the mutation (58). The development of drugs that target events downstream of spliceosomal mutations provides a viable strategy for the treatment of SF3B1 mutant MDS.

## 5 Prognostic and predictive markers in patients with SF3B1 mutated MDS

### 5.1 Prognosis

The International Working Group on Prognosis of MDS (IWG-PM) recently proposed diagnostic criteria for SF3B1 as a separate entity in MDS, including (a) cytopenia as defined by standard hematologic values, (b) somatic SF3B1 mutations, (c) morphologic dysplasia (with or without RS), and (d) bone marrow blasts <5% and peripheral blood blasts <1%. Concomitant genetic disorders became exclusion criteria for the proposed entity. Their study suggests that MDS with SF3B1 mutations has a slower course and may serve as a specific diagnostic subtype with an improved prognosis (7).

It has been shown earlier that the association of SF3B1 mutations with good prognosis correlates with the better function of MDS-RS, while the mutations themselves do not have an independent prognostic correlation (93, 94). However, most of the findings are consistent with the IWG-PM, showing that SF3B1 mutations are associated with better clinical performance and prognosis in MDS (5, 23, 36, 76, 90, 95). Which may be strongly associated with higher age, higher platelet (Plt) counts, lower red blood cell (RBC) counts, higher white blood cell (WBC) counts, common RS in the bone marrow, lower maternal cell levels, lower International Prognostic Scoring System (IPSS) risk, and longer OS.

The incidence of treatment-associated MDS (t-MDS), which has a poor response to treatment and a high-risk profile, is increasing every year (96). Volpe et al. compared *de novo* SF3B1 mutant MDS (SF3B1^mut^ *de novo* MDS) with SF3B1 mutant t-MDS (SF3B1^mut^ t-MDS), SF3B1^mut^ t-MDS with SF3B1 wild-type t-MDS (SF3B1^wt^ t-MDS) groups were compared and found that even in the case of t-MDS, MDS with SF3B1 mutation was suggestively associated with a good prognosis in terms of median OS as well as cytogenetics, validating the prognostic situation of MDS-SF3B1 proposed by IWG-PM in t-MDS (97).

### 5.2 Predictive markers

The prognostic impact of SF3B1 mutations may vary among different MDS entities, reflecting differences in replicative stress-driven tumorigenesis or differential genomic stability and cell viability of different cell types after treatment (58, 98, 99). Prognostic factors affecting MDS with SF3B1 mutations are closely related to the disease phenotype and the treatment modality received. It has been shown that SF3B1 mutations favor MDS-RS-SLD, MDS-RS-MLD, and MDS-EB-2 (the most significant favorable effects were found, especially in MDS- RS-SLD). In MDS with del(5q) syndrome, SF3B1 mutations were associated with shorter OS. Blast count <5% as a promising prognostic marker, and MDS with excess blasts were associated with the shortest OS. Notably, 3/12 MDS-EB-2 patients with SF3B1 mutation in this study underwent allogeneic HSCT (9).

At least 40% of patients with MDS have at least two mutations (8, 100). There are data suggesting that concurrent mutations are present in more than 80% of patients with MDS with SF3B1 mutations in patients with very low/low and intermediate risk MDS. Common concurrent mutations are TET2, DNMT3A, SRSF2, CDH23, ASXL, CUX1, and KMT2D (36, 101). The presence of concurrent mutations can alter the good prognosis of patients with SF3B1 mutated MDS. Mutations in TP53, RUNX1, ASXL1, SRSF2, IDH2, BCOR, STAG2, and NUP98 have been described as poor prognostic markers in concomitant SF3B1 mutated MDS. Their co-occurrence with SF3B1 may have worse OS and higher poor prognostic value associated with AML conversion rates (9, 74, 102, 103). Moreover, the number of concurrent mutations was also positively correlated with patient survival, with patients with at least two mutations accompanying SF3B1 having a greater detrimental effect on survival (75).

The identification of SF3B1 mutation hotspot types is essential for risk stratification. Kanagal- Shamanna et al. showed that about 40% of SF3B1 mutation MDS cases showed non-K700E mutations, and further classification studies found that only SF3B1 mutation MDS patients with involvement of K700E mutations had a good prognosis. In contrast, non-K700E mutation SF3B1-mutated MDS patients had a similar prognosis to that of MDS patients without SF3B1 mutations, with the only significant adverse clinical feature being a lower neutrophil count. Mutations involving the K700E hotspot independently predict OS in SF3B1 mutant MDS patients (25).

Thus, patients with MDS with SF3B1 mutations may represent a subset of good prognosis provided that other poor prognostic markers are excluded (9). In addition, there may also be relevant evidence that can be extracted based on the pathways associated with splicing features, suggesting prognostic information for SF3B1 mutant MDS patients that can be further developed as biomarkers for risk stratification (13) (**Supplementary Figure 2**).

## 6 Conclusion

The current high incidence of splicing factor mutations in MDS has stimulated interest in their tumor-driving mechanisms and in targeting splicing mutation therapy. A growing body of evidence suggests that SF3B1 mutations have great potential in diagnosis, prognosis, and in the treatment of MDS. With the development of next-generation sequencing technology, the molecular biological characterization of SF3B1 has become a current research hotspot. Several studies are underway to further clarify the driving mechanism of SF3B1 in MDS. Existing studies on the pathophysiological mechanisms of SF3B1 mutations in MDS have focused on impaired erythropoiesis, iron metabolism disorders, hyperinflammatory features, and R-loop accumulation, which constitute the significant clinical features of MDS. Although most findings suggest a relatively good prognosis in MDS-RS patients with SF3B1 mutations, the prognosis in MDS patients containing poor predictors remains unsatisfactory, and effective biomarkers to guide their prognostic significance remain to be elucidated in clinical application.

Currently, MDS is treated mainly with supportive, demethylation, and immunosuppressive therapy. However, there remain significant unmet medical needs in MDS that require developing new therapeutic approaches. The genetic and biological heterogeneity of MDS presents opportunities and challenges for developing new clinical treatments. Many studies are exploring emerging therapeutic strategies at the molecular level are being explored that promise to be more refined and targeted, leading to better outcomes and improved quality of life for patients.

Scientists are exploring novel drugs that target the molecular pathways involved in the pathogenesis of MDS. Newly discovered small molecules show great promise as potential inhibitors of cellular processes in managing heterogeneous hematological malignancies. As mechanistic studies mature, additional targets at the splicing factor level are expected to emerge. Targeting clonal cells of MDS by RNA splicing is a future trend with great potential.

RNA splicing is an essential process that occurs in all eukaryotes. We can develop more effective and personalized therapies for patients with MDS by targeting splicing. Studying individualized splice gene expression, identifying available mutant gene targets at the preclinical stage, and addressing the challenges of drug resistance and ineffectiveness will help us better serve patients with MDS and other diseases.

In conclusion, SF3B1, as the most frequently occurring splicing factor mutation in MDS, is involved in all stages, from diagnosis to prognosis to targeted therapy. Although the drugs on targeting splicing have not been fully overcome, with further studies on the pathophysiological mechanisms of SF3B1 splicing factor mutations in malignant hematologic tumors, systematic characterization and evaluation of the splicing events generated by these mutations provide highly informative insights from diagnosis to prognosis to targeted therapy.SF3B1, as a potential target for MDS treatment, will give practical aid for future clinical work.

## Author contributions

MJ wrote of original manuscript. WZ, MC, QL and XY took part in the manuscript modification. ZJ helped with the preparation of the figure. All authors contributed to the article and approved the submitted version.
