# Supplementary material for: SF3B1 mutations in myelodysplastic syndromes: A potential therapeutic target for modulating the entire disease process
Source: Front Oncol. 2023 Mar 17;13:1116438. doi: 10.3389/fonc.2023.1116438 (PMC10063959; doi:10.3389/fonc.2023.1116438)
Supplement: Supplementary file 1 [file DataSheet_1.pdf]

Supplementary Material

SF3B1 mutations in myelodysplastic syndromes: a potential therapeutic target for modulating the entire disease process

Moqin Jiang[1,2,3],Meng Chen[1,2],Qian Liu[1,2],Zhiling Jin[1,2,3],Xiangdong Yang[1,2],Weifeng Zhang[1,2*]

[1] Department of Hematology, First Teaching Hospital of Tianjin University of Traditional Chinese Medicine, Tianjin, China

[2] National Clinical Research Center for Chinese Medicine Acupuncture and Moxibustion, Tianjin, China

[3] Graduate School, Tianjin University of Traditional Chinese Medicine, Tianjin, China

*** Correspondence:**
Weifeng Zhang*
wf031@163.com

Supplementary Figures

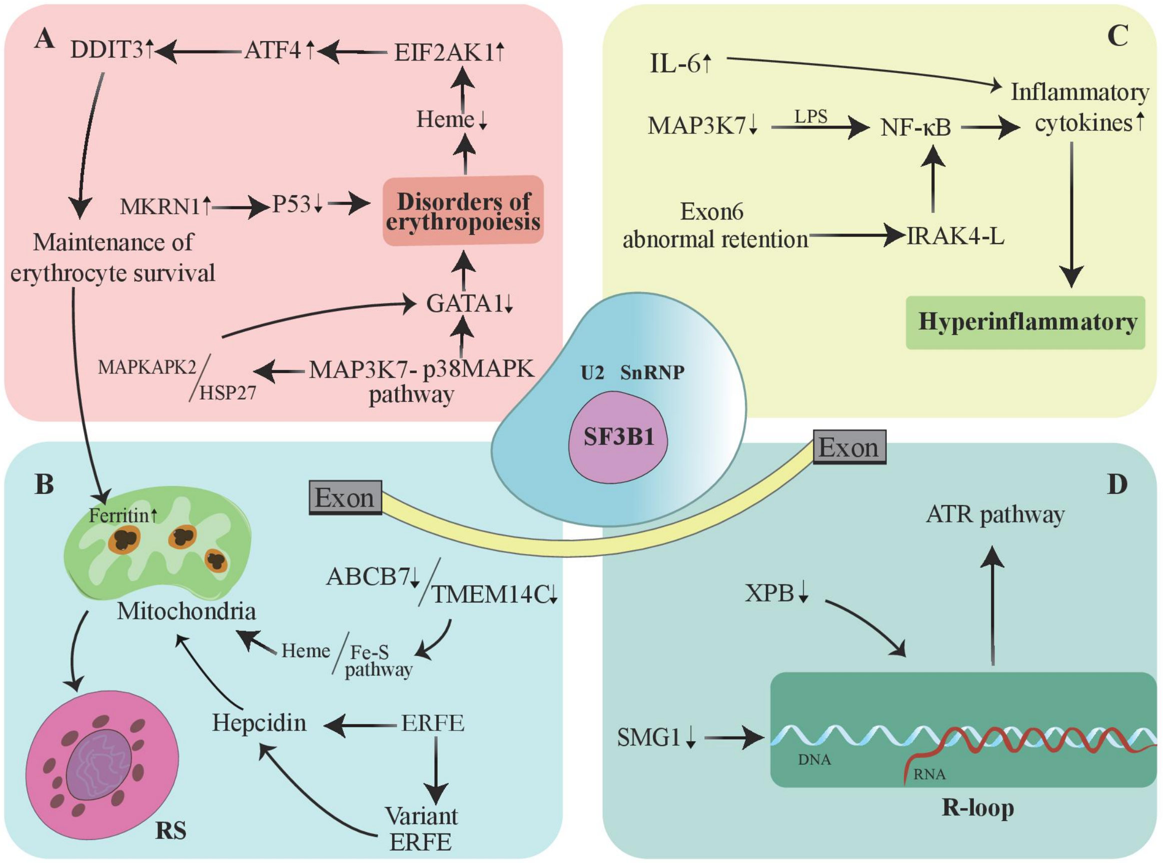

Figure 1. SF3B1 can affect MDS pathogenesis through the following pathways.

(A) Misspliced serine/threonine protein kinase MAP3K7 disrupts the MAP3K7-p38MAPK pathway, inactivating its potential downstream targets MAPKAPK2 and HSP27. Early downregulation of GATA1, a key regulator of erythroid differentiation, affects erythroid proliferation, differentiation, and, ultimately, apoptosis; Decreased binding of the metabolic stress-sensitive kinase EIF2AK1 to heme, resulting in increased expression of the stress response effector ATF4 and its downstream effector DDIT3 to maintain the survival of red lineage cells in the absence of heme and possibly control iron overload due to abnormal mitochondrial function; A reduction in the large isoform of MKRN1, a transcriptional co-regulator, and an E3 ligase, downregulates protein levels of P53 and P53 downstream targets, which may trigger increased apoptosis and cell cycle arrest triggered.

(B) The expression of iron homeostasis regulators ABCB7 and TMEM14C was down-regulated and acted synergistically. Affected heme and Fe-S pathways induced significant upregulation of ferritin in mitochondria, disrupting the sequence of heme synthesis, reducing the erythrocyte set-apart capacity of normal bone marrow cells, and leading to RS formation; ERFE transcription generated a variant protein and, together with canonical transcripts, led to ERFE overexpression, which inhibited hepcidin transcriptional function and disturbed iron content, tissue distribution, and iron supply to erythropoiesis in the organism.

(C) Downregulation of the inflammatory cytokine IL-6, which activates NF-κB signaling and abnormalizes the hematological profile of MDS patients; MAP3K7 downregulation and imposition of the Toll-like receptor (TLR) agonist lipopolysaccharide (LPS) stimulation revealed that NF-κB signaling was hyperactivated; IRAK4 is a key downstream mediator that closely links the

Myddosome complex to inflammatory NF-κB activation, and SF3B1 mutations resulting in exon six being aberrantly retained in MDS samples produced a longer long IRAK4-long (IRAK4-L) isoform, which maximizes activation of downstream NF- kB signaling.

(D) Toxic protein products formed by SMG1, a core kinase that activates the NMD machinery in animals, may lead to R-loop accumulation, further leading to impaired DNA replication, DNA damage, chromosome instability, and cell death; Loss of XPB activity, an essential component of the encoded transcription factor IIH (TFIIH) with eukaryotic transcriptional and DNA repair roles, promotes R-loop-mediated DNA damage. The accumulation of R-loop and associated DNA damage due to SF3B1 mutation activates the ATR pathway involved in DNA repair in MDS cells.

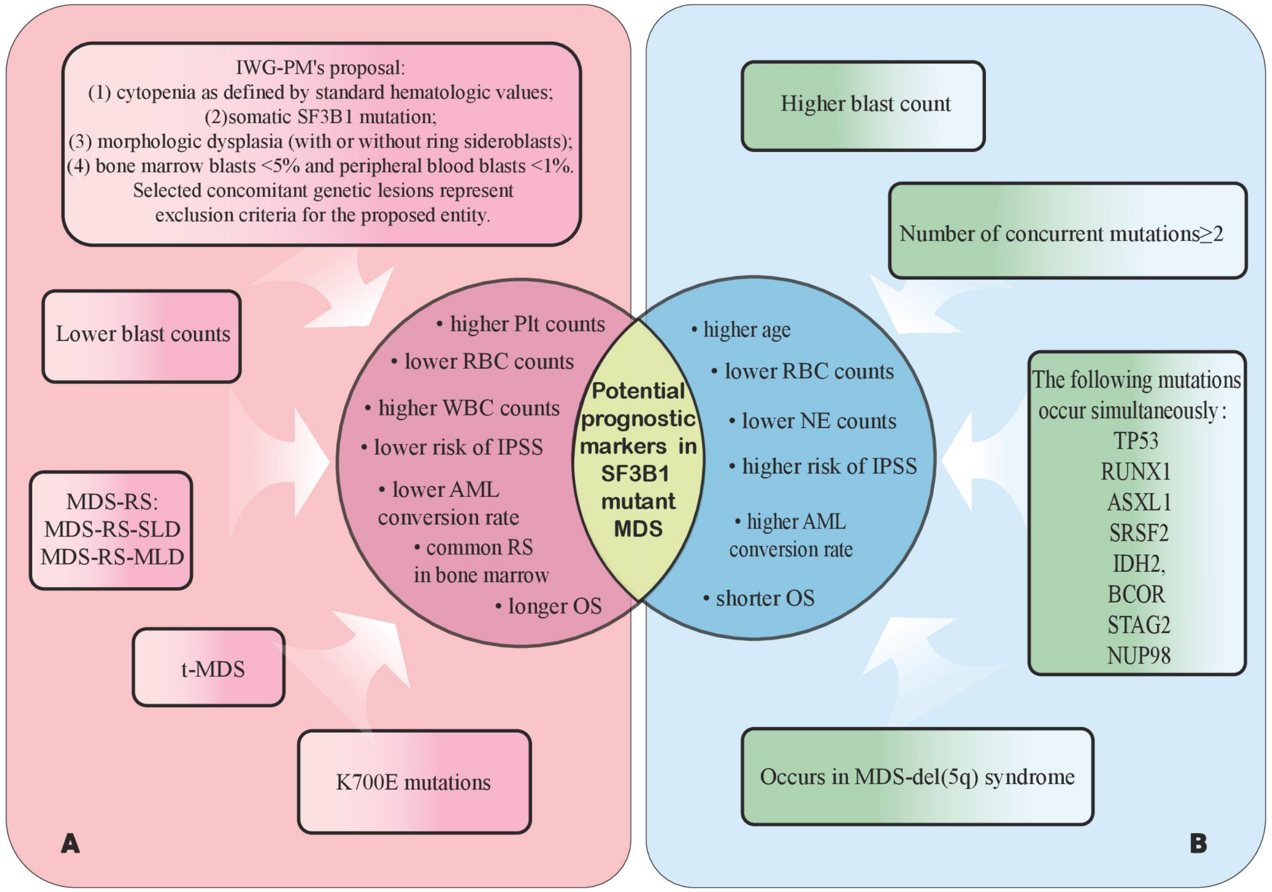

Figure 2. Different diagnostic criteria for MDS, blast levels, mutation sites, treatment options, and predictors of concurrent mutations potentially affect the prognostic level of SF3B1 mutated MDS.

Table 1. Summary of Researches on Targeted SF Spliceosome Inhibitors.

Spliceosome inhibitor	Ingredient	Mechanism	Clinical Trial	Objective	Diseases targeted	Security	Therapeutic effect	References
**E7107**	Derivatives of pladienolide B	Disrupting the assembly of U2 snRNP at the 3 ' splice site.	Phase I	Determine the maximum tolerated dose, dose-limiting toxicity and pharmacokinetic profile of E7107.	Metastatic or locally advanced solid tumors	Optic nerve damage.	E7107 was well tolerated and the clinical study was terminated after the occurrence of optic nerve damage.	(82, 87)
**meayamycin**	Derivatives of FR901464	Interferes with SF3B at an early stage and prevents the assembly of in vitro spliceosomes.	No	Clarification of the picomolar antiproliferative activity of meayamycin against various cancer cell lines and multidrug resistant cells.	Breast cancer, colon cancer, lung cancer, prostate cancer, cervical cancer	-	-	(84)
**Jerantinine A (JA)**	Indole alkaloids	Inhibits microtubule protein polymerization and induces G2/M cell cycle arrest and tumor-specific cell death.	No	Investigating the pathways by which JA exerts its antitumor activity	Breast Cancer	-	-	(85)
**spliceostatin A (SSA)**	Methylated derivatives of FR901464	Weakened the ability of SF3B to bind RNA and induced it to attach to the "decoy" sequence.	No	Clarify the pharmacological mechanism of SSA.	-	High cytotoxicity for multiple cancer models.	-	(88)
**GEX1A**	Derivatives of Herboxidiene	Interference between SF3B1 and the PHF5A subunit of U2 snRNP.	No	Assessing the molecular mechanism GEX1A kills leukemic cells in vitro and within in vivo mouse models.	Leukemia	-	-	(87)

**H3B-8800**	Derivatives of pladienolide	Binding SF3B1 inhibits aberrant splicing of TMEM14C in MDS patients.	Phase I	Evaluation of the antitumor activity of H3B-8800 in patients with myeloid neoplasms.	MDS, CMML ,AML	Security is under control.	No complete or partial responses meeting IWG criteria were observed.	(86, 91)
**OTS964**	Highly selective CDK11 inhibitor	Binding to SF3B1 and phosphorylation of its threonine residues during spliceosome activation.	No	Identification of CDK11 as responsible for phosphorylation of SF3B1 during spliceosome activation.	-	-	-	(92)
